# Feasibility of an alternative, physiologic, individualized open-lung approach to high-frequency oscillatory ventilation in children

**DOI:** 10.1186/s13613-019-0492-0

**Published:** 2019-01-18

**Authors:** Pauline de Jager, Tamara Kamp, Sandra K. Dijkstra, Johannes G. M. Burgerhof, Dick G. Markhorst, Martha A. Q. Curley, Ira M. Cheifetz, Martin C. J. Kneyber

**Affiliations:** 1 Department of Paediatrics, Division of Paediatric Critical Care Medicine, Beatrix Children’s Hospital, University Medical Center Groningen, University of Groningen, Huispost CA 80, P.O. Box 30.001, 9700 RB Groningen, The Netherlands; 2 Department of Epidemiology, University Medical Center Groningen, University of Groningen, Groningen, The Netherlands; 3 Department of Paediatrics, Division of Paediatric Critical Care Medicine, VU University Medical Center, Amsterdam, The Netherlands; 4 Family and Community Health, School of Nursing, Anesthesia and Critical Care Medicine, Perelman School of Medicine, University of Pennsylvania, Philadelphia, PA USA; 5 Department of Pediatrics, Division of Critical Care Medicine, Duke University School of Medicine, Durham, NC USA; 6 Critical Care, Anaesthesiology, Perioperative and Emergency Medicine (CAPE), University of Groningen, Groningen, The Netherlands

**Keywords:** Acute respiratory failure, Paediatric acute respiratory distress syndrome, High-frequency oscillatory ventilation, Mechanical ventilation, Paediatrics, Child, Oxygenation

## Abstract

**Background:**

High-frequency oscillatory ventilation (HFOV) is a common but unproven management strategy in paediatric critical care. Oscillator settings have been traditionally guided by patient age and/or weight rather than by lung mechanics, thereby potentially negating any beneficial effects. We have adopted an open-lung HFOV strategy based on a corner frequency approach using an initial incremental–decremental mean airway pressure titration manoeuvre, a high frequency (8–15 Hz), and high power to initially target a proximal pressure amplitude (∆*P*_proximal_) of 70–90 cm H_2_O, irrespective of age or weight.

**Methods:**

We reviewed prospectively collected data on patients < 18 years of age who were managed with HFOV for acute respiratory failure. We measured metrics for oxygenation, ventilation, and haemodynamics as well as the use of sedative-analgesic medications and neuromuscular blocking agents.

**Results:**

Data from 115 non-cardiac patients were analysed, of whom 53 had moderate-to-severe paediatric acute respiratory distress syndrome (PARDS). Sixteen patients (13.9%) died. Frequencies≥ 8 Hz and high ∆*P*_proximal_ were achieved in all patients irrespective of age or PARDS severity. Patients with severe PARDS showed the greatest improvement in oxygenation. pH and PaCO_2_ normalized in all patients. Haemodynamic parameters, cumulative amount of fluid challenges, and daily fluid balance did not deteriorate after transitioning to HFOV in any age or PARDS severity group. We observed a transient increase neuromuscular blocking agent use after switching to HFOV, but there was no increase in the daily cumulative amount of continuous midazolam or morphine in any age or PARDS severity group. No patients experienced clinically apparent barotrauma.

**Conclusions:**

This is the first study reporting the feasibility of an alternative, individualized, physiology-based open-lung HFOV strategy targeting high *F* and high ∆*P*_proximal_. No adverse effects were observed with this strategy. Our findings warrant further systematic evaluation.

## Introduction

High-frequency oscillatory ventilation (HFOV) is considered as a rescue intervention in children suffering from acute lung injury when conventional mechanical ventilation (CMV) fails [1, 2]. However, there are scarce paediatric scientific data supporting its use. The only paediatric randomized controlled trial (RCT) that evaluated the effects of HFOV on patient outcome was published in 1994 and found no differences in mortality rates between HFOV and CMV [3]. Continued use of HFOV in paediatrics became even more controversial following two negative clinical trials in adult ARDS and two recent paediatric observational reports failing to show any benefit of HFOV on patient outcome [4–7]. Gupta et al. reported increased mortality and prolonged duration of mechanical ventilation associated with early or late HFOV in a database analysis study using propensity score matching, although there were serious methodological issues related to this study [6, 8, 9]. Bateman and co-workers performed a post hoc analysis of paediatric patients enrolled into a large protocolized paediatric sedation trial using propensity matching with clinically relevant variables [7]. When patients managed with early HFOV were compared with those supported with late HFOV or CMV, mortality was unchanged, but duration of mechanical ventilation was prolonged in the early HFOV group.

It remains unclear whether the outcomes of these studies confirm that HFOV is not beneficial, or even harmful, or that patient outcome was determined by the oscillator management strategy [10–12]. Most paediatric HFOV studies make no mention of recruitment manoeuvres (RM) and reported frequencies (*F*) in the range of 5–8 Hz. Yet, optimizing lung volume by means of a RM seems physiologically necessary when transitioning to HFOV to recruit collapsed, atelectatic lung units being exposed to larger, potentially more injurious pressure swings [13]. Furthermore, use of low oscillatory *F* is not in line with the concept of the corner frequency (*Fc*) [2]. *Fc* is the *F* at which the pressure cost of ventilation is the lowest, in other words the *F* that is the least injurious to the lung [14]. In disease conditions with reduced respiratory system compliance (Crs), such as ARDS, *Fc* is increased indicating that the highest oscillatory *F* that still allows for adequate ventilation might be preferable.

We started using an individualized, physiology-driven strategy to HFOV as an alternative mode of ventilation for PARDS rather than the more traditional size/age-based approach as a rescue intervention. Our strategy is centred on an open-lung concept using an initial staircase incremental–decremental mean airway pressure (mPaw) titration manoeuvre, a high *F* (12 Hz), and high power to initially target a proximal pressure amplitude (∆*P*_proximal_) of 70–90 cm H_2_O, irrespective of age or weight. Here, we report our first experiences with this alternative approach to HFOV in a heterogeneous group of patients with moderate-to-severe acute lung injury. We studied the feasibility of our strategy and examined the level and time course of metrics for oxygenation and ventilation and hemodynamic parameters as well as the use of sedative-analgesic medications and neuromuscular blocking agents (NMBA).

## Methods

### Study design and setting

This study was designed as a retrospective review of prospectively collected data between January 2013 and December 2015.

### Patients

Included were all children younger than 18 years of age managed with HFOV for acute respiratory failure originating from any cause, defined by acute onset, presence of ≥ 1 infiltrate on chest radiograph, PaO_2_/F_I_O_2_ < 300 mmHg and PEEP ≥ 5 cm H_2_O. Patients with status asthmaticus, upper airway disorders, and underlying (congenital) cardiac anomalies were excluded. All patients had body weight within appropriate for age.

### Data collection

Demographic, physiological, laboratory and ventilator parameters were manually extracted from the patient’s medical record. Disease severity was assessed by the Pediatric RIsk of Mortality (PRISM) III 24-h score. We applied the paediatric ARDS (PARDS) definition to identify patients with ARDS [15]. All consecutive PICU chest radiograph images were reviewed by a paediatric radiologist to determine the presence or absence of pulmonary infiltrates.

### Variable definition and calculation

Physiological and laboratory data included heart rate, invasively measured arterial systolic, mean and diastolic blood pressure (mABP), central venous pressure (if a central line was in situ), and transcutaneous measured oxygen saturation (SpO_2_). These variables were continuously monitored using a Philips MP70 Intellivue monitor (Philips Medical Systems, Best, the Netherlands) and documented hourly by the bedside nurse. Although the frequency of arterial blood sampling was completed at the discretion of the attending physician, typically, arterial blood gases and lactate are measured at least every 6 h daily in the early phase of the oscillatory trajectory unless the clinical condition of the patient warranted more frequent analysis (Radiometer, Brønshøj, Denmark). Ventilator parameters for conventional mechanical ventilation (CMV) included PIP, mean airway pressure (mPaw), positive end-expiratory pressure (PEEP), expiratory tidal volume (Vte), and FiO_2_; for HFOV, these included mPaw, *F*, ∆*P* and FiO_2_. Vte was measured near the Y-piece of the endotracheal tube in patients < 10 kg (VarFlex™, Vyaire, Yorba Linda, CA, USA). These data were documented hourly. The PF ratio and oxygenation index (OI: mPaw * FiO_2_ * 100)/PaO_2_) were computed using concurrent blood gas and ventilator data.

To study haemodynamics, we analysed the daily cumulative fluid intake and number of fluid boluses administered. A daily vasoactive inotrope score (VIS) was calculated to describe the need for vasoactive support [16]. The non-respiratory Pediatric Logistic Organ Dysfunction (PELOD) 2 score was calculated daily to describe organ dysfunction [17]. The use of neuromuscular blocking agents (NMBAs) was noted as well as total daily cumulative dosage of sedatives and analgesic drugs. The use of NMBA, sedation and analgesia were managed using a unit-based clinical algorithm.

### CMV protocol

Patients were managed per a unit-based algorithm. This algorithm prescribes the use of a time-cycled, pressure-limited ventilation mode (pressure control (PC)/assist control (AC) in children < 12 months or PC/synchronized intermittent mandatory ventilation (SIMV) in children ≥ 12 months) in children with acute lung injury. Expiratory tidal volume (Vte) is measured near the Y-piece of the endotracheal tube (ETT) in children < 10 kg (VarFlex™, Vyaire, Mettawa, Ill, USA). We target PIP < 30–32 cm H_2_O and maximum Vte 5–8 mL/kg actual bodyweight in all patients. Initial PEEP at the start of CMV is 4–6 cm H_2_O in all patients, adjustments are dictated by the FiO_2_ at the discretion of the attending physician. We do not use the ARDS Network PEEP/FiO_2_ grid or lung volume optimization manoeuvres such as staircase PEEP titration or sustained inflation during CMV [18]. Mandatory breath rate is dictated by underlying respiratory mechanics and age to maintain pH within target range; the flow-time scalar is carefully observed to prevent auto-PEEP. The maximum I/E ratio is 1:1. The amount of pressure support in the PC/SIMV mode is calculated by PIP minus PEEP.

### HFOV protocol

Patients are oscillated per a unit-based algorithm that defines HFOV criteria, recruitment manoeuvre (RM), and titration of HFOV settings according to the evolving physiologic needs of each patient (SensorMedics 3100; Vyaire, Yorba Linda, CA, USA). Transitioning to HFOV is performed when peak inspiratory pressure (PIP) > 28–32 cm H_2_O, PEEP > 8 cm H_2_O, FiO_2_ > 0.60, and oxygenation index (OI) increases on three consecutive 1-hour measurements despite increasing PEEP and using neuromuscular blockade. A specific OI or mPaw threshold is not used to initiate HFOV. Vt was not measured when the patient was on HFOV.

**Fig. 1:**
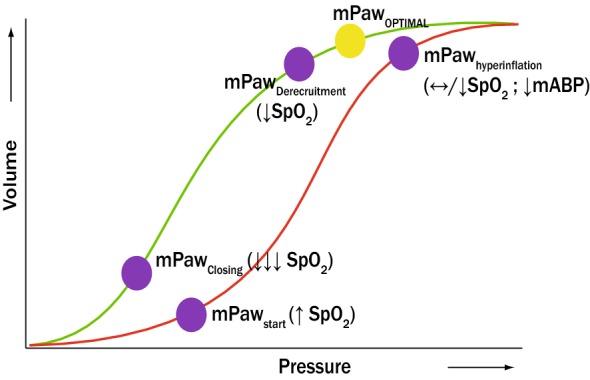
Graphical simplification of the stepwise incremental–decremental mPaw titration when switching to HFOV. The red line represents the inspiratory limb of the pressure volume loop, whereas the green line represents de deflation limb. The mPaw is increased by 2 cmH_2_O every 3–5 min until no further improvement in SpO_2_ and/or decrease in mean ABP occurs during two consecutive increments (identifying mPaw_recruitment_ and mPaw_hyperinflation_). Then, the mPaw is decreased by cmH_2_O every 3–5 min until SpO_2_ decreased (mPaw_derecruitment_) during two consecutive decrements. The RM was repeated to mPaw_hyperinflation_ with setting the “optimal” mPaw + 2 cm H_2_O above mPaw_derecruitment_. mPaw mean airway pressure; SpO_2_ transcutaneously measured oxygen saturation; mABP mean arterial blood pressure

**Fig. 2:**
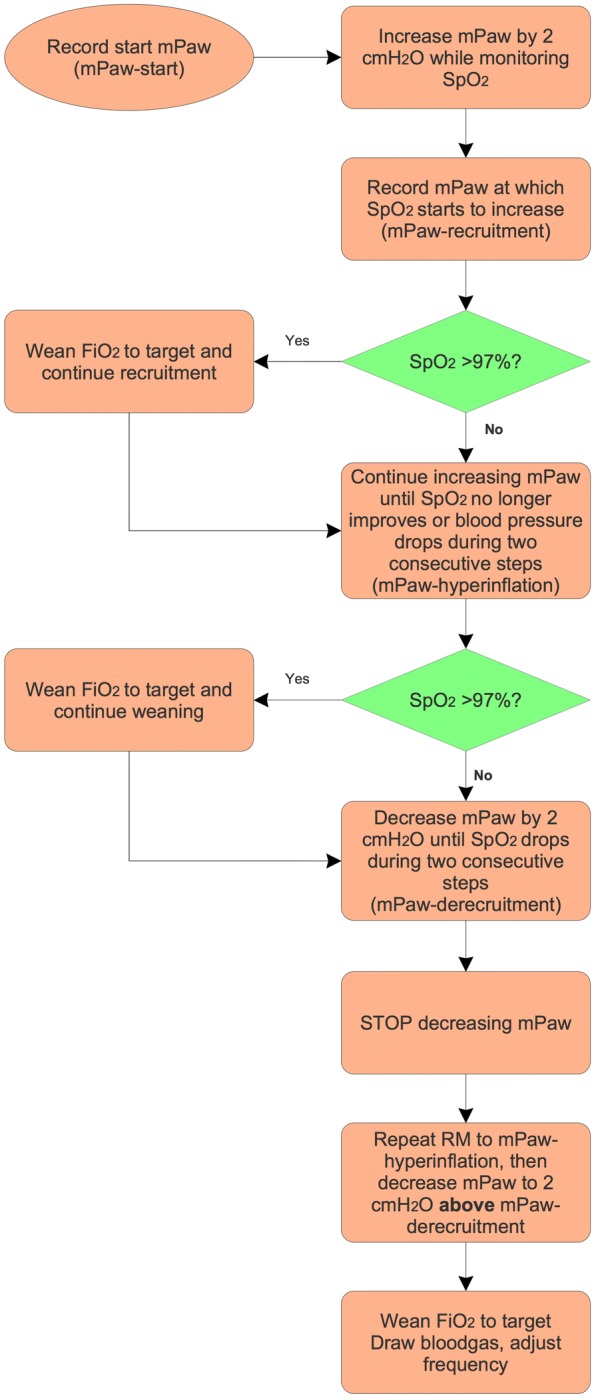
HFOV stepwise incremental–decremental mPaw titration

The following starting HFOV settings are used: *F* 12 Hz, mPaw 3 cm H_2_O above mPaw on CMV, ∆*P*_proximal_ 70–90 cm H_2_O, inspiratory time 33%, and bias flow 20–40 L/min, irrespective of age or bodyweight. Immediately after switching to HFOV, we perform an individualized staircase incremental–decremental mPaw titration to find the optimal initial mPaw on the deflation limb of the pressure–volume relation (Figs. 1, 2). In brief, immediately after transitioning to HFOV, we perform an individualized staircase incremental–decremental mPaw titration to find the optimal initial mPaw on the deflation limb of the pressure–volume relation (Fig. 1). First, we increase the mPaw 2 cm H_2_O every 3–5 min while simultaneously observing the SpO_2_ (as proxy for lung volume) and mean ABP until no further improvement in SpO_2_ and/or decrease in mean ABP occurs during two consecutive increments. This allows us to identify the onset of lung recruitment (i.e. increase in SpO_2_, mPaw_recruitment_) and the onset of lung overdistension (mPaw_hyperinflation_). If during the RM the SpO_2_ exceeds 97%, we reduce FiO_2_ and continue the RM. Next, we decrease the mPaw by 2 cm H_2_O every 3–5 min until SpO_2_ decreases (mPaw_derecruitment_) during two consecutive decrements. The RM is repeated to mPaw_hyperinflation_ with setting the “optimal” mPaw + 2 cm H_2_O above mPaw_derecruitment_. During the RM, oscillations are continued.

The RM is discontinued in the event of bradycardia and/or refractory hypotension (i.e. > 20% decrease in mABP for > 5 min). Chest radiographs are not routinely taken after the RM. During ongoing support, the mPaw is actively decreased by 2 cm H_2_O if FiO_2_ < 0.40–0.50 and SpO_2_ is within target range. *F* is decreased by 0.5–1.0 Hz (min 8 Hz) when the pH is below target range and increased by 0.5–1 Hz if the pH rises above target range. We decrease the power by 10% if hypocapnia occurs at *F* 15 Hz. The ETT cuff is routinely inflated.

Failure of HFOV is defined by the inability to wean either the mPaw or the FiO_2_ over the first 24 h following start of HFOV or if there is a worsening of the oxygenation index despite “maximum” HFOV settings. If a patient meets these criteria, he will be cannulated for extra-corporeal life support (ECLS).

### Targets of oxygenation and ventilation in all patients

FiO_2_ is adjusted to maintain SpO_2_ 88–92%. Unless dictated by the clinical condition, permissive hypercapnia is allowed targeting pH ≥ 7.20 irrespective of PaCO_2_. Transcutaneous CO_2_ monitoring is not used.

### Supportive care in all ventilated patients

All patients were ventilated in the supine position and received continuous intravenous infusion of analgesic-sedative drugs, including midazolam and morphine. The bedside nurse titrated the analgesic-sedative drugs guided by the heart rate and pupils (for patients on neuromuscular blockade) or by the Comfort B score (for non-paralyzed patients) [19]. Hemodynamic management of ventilated patients included targeting a fluid nil balance via fluid restriction (± 75% of normal fluid intake) and intravenous diuretic therapy (continuous intravenous administration of furosemide). The decision to prone a patient is at the discretion of the attending physician.

### Study endpoints

The primary endpoint of this study was feasibility of our protocol (in each patient, defined as maintaining *F* ≥ 8 Hz and ∆*P*_proximal_ 70–90 cmH_2_O as function of disease severity. Secondary endpoints included the level and trajectory of metrics for oxygenation and ventilation as function of disease severity and the level and trajectory of hemodynamic parameters, occurrence of new air leak, daily cumulative dosage of sedative-analgesic drugs and use of NMBA, and level and time course of the non-respiratory PELOD-2 as function of age. The latter was chosen because the current approach to HFOV calls for oscillator settings dictated by age and/or weight.

### Statistical analysis

Continuous data are presented as median and 25–75 interquartile range (IQR) since assumptions of normality were not always satisfied. Categorical data are presented as percentage (%) of total. When comparisons between groups were made, continuous data were analysed using the Kruskal–Wallis test, and the Chi-square test with Yates continuity correction was used to analyse categorical data. Generalized linear model analyses were performed to analyse the effects of PARDS severity, age, survival status and time as well as the interaction between time and age or PARDS severity on all study endpoints as these parameters are repeated measurements. All statistical analyses were performed using SPSS for Mac (IBM, Chicago, Ill, USA). *p* values below 0.05 were considered statistically significant.

## Results

### Patient characteristics

During the 3-year study period, 1787 patients were mechanically ventilated > 24 h (58.2% of all admitted patients). One hundred and forty-eight (8.3%) of those patients were supported on HFOV. Eleven patients were excluded from analysis because they were oscillated for status asthmaticus (*N* = 6) or upper airway disorders (*N* = 3) or had an incomplete dataset (*N* = 2). Twenty-two (16.1%) patients were excluded due to congenital heart lesions. Thus, data from 115 patients were eligible for analysis.

Table 1 summarizes baseline patient characteristics and last ventilator settings prior to HFOV stratified by PARDS severity. The three groups were well-balanced except for the PRISM-III 24-h score. The majority of patients had direct lung injury. No patients were proned. Two patients were cannulated for ECMO: one with moderate PARDS and the other with severe PARDS. Sixteen (13.9%) patients died during their PICU stay; mortality was the highest (33.3%) in severe PARDS patients. Eight patients died because treatment was redirected based on the underlying disorder (e.g. chronic lung disease (*N* = 4), neuromuscular disorders (*N* = 1) or haemato-oncologic disorder (*N* = 3); seven patients died of multiple organ failure. Except for one drowning victim, all patients had serious co-morbidities. No patient died from refractory hypoxemia.

**Table 1:** Study population characteristics and last ventilator settings prior to high-frequency oscillatory ventilation

Variable	No or mild PARDS (*N* = 62)	Moderate PARDS (*N* = 29)	Severe PARDS (*N* = 24)
Age (months)	4.5 (2.0–12.5)	5.0 (2.0–24.5)	6.0 (1.8–20.23)
0–12 months (%)	75.8	69.0	66.7
13–60 months (%)	14.5	20.7	20.8
> 60 months (%)	9.7	10.3	12.5
Male (%)	54.8	48.3	45.8
Weight (kg)	7.0 (4.5–10.8)	5.5 (3.8–11.5)	6.9 (4.1–11.3)
PRISM-III 24 h*	6.0 (3.0–8.0)	6.0 (3.0–6.5)	12.5 (8.23–37.78)
Admission diagnosis (%)			
Pulmonary	85.5	79.3	79.2
PHT	1.6	0	8.3
Acute liver failure	0	0	8.3
Pancreatitis	1.6	0	0
Sepsis	9.7	13.8	4.2
Post-operative	1.6	6.9	0
PICU stay (days)	10.0 (7–17)	10.0 (8.0–15.0)	12.5 (8.3–37.8)
PICU mortality (%)*	6.5	10.3	33.3
*Last ventilator settings on conventional mechanical ventilation before switch to HFOV*			
PIP (cmH_2_O)	29 (27–32)	28 (26–30)	29 (27–33)
mPaw (cmH_2_O)	15 (14–16)	16 (14–17)	17 (15–19)
PEEP (cmH_2_O)	7.0 (6.0–8.1)	7.8 (6.5–8.1)	7.4 (6.7–9.9)
Vt_exp_ (mL/kg)	6.3 (5.7–7.5)	7.0 (6.3–7.9)	7.0 (6.2–8.0)
FiO_2_*	0.61 (0.50–0.99)	0.75 (0.55–0.90)	0.99 (0.75–1.0)
Time on CMV before start HFOV (h)	10 (3–14)	10 (5–17)	11 (3–16)
Total length of HFOV run (h)	91 (66–123)	102 (67–149)	134 (69–224)
Total length of CMV run after HFOV (h)	72 (28–160)	73 (12–169)	77 (22–172)

Data are depicted as median (25–75 interquartile range) or percentage (%) of total

PARDS pediatric acute respiratory distress syndrome; *PRISM* pediatric risk of mortality, *PHT* pulmonary hypertension, *PICU* pediatric intensive care unit, *PARDS* pediatric acute respiratory distress syndrome, *PIP* peak inspiratory pressure, *PEEP* positive end-expiratory pressure, *Vt* tidal volume, *CMV* conventional mechanical ventilation, *HFOV* high-frequency oscillatory ventilation

*p < 0.05

### Level and time course of *F*, ∆*P*_proximal_ and mPaw as function of severity

**Fig. 3:**
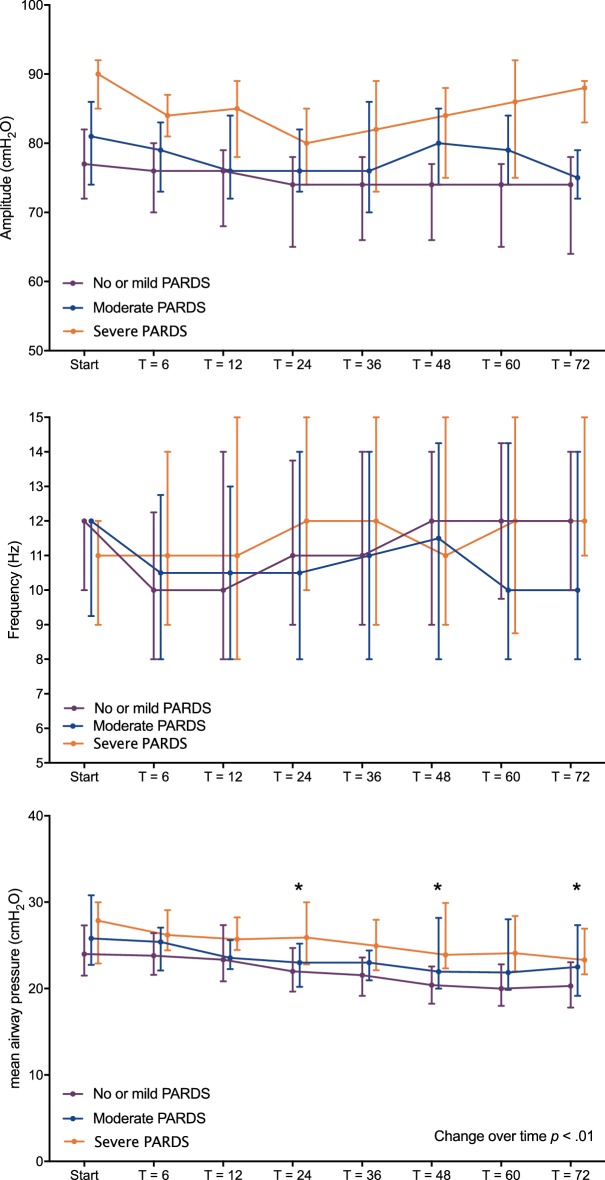
Level and time course of achieved, frequency (*F*) (upper panel), proximal pressure amplitude ∆*P*_proximal_ (middle panel) and mean airway pressure (mPaw) (lower panel)during the first 72 h of high-frequency oscillatory ventilation (HFOV), stratified by paediatric acute respiratory distress syndrome (PARDS) severity (*N* = 62 children no/mild PARDS, *N* = 29 children moderate PARDS, *N* = 24 children severe PARDS). “Start” is the first measurement immediately after the recruitment manoeuvre. Data are depicted as median (25–75 interquartile range). *p < 0.05

Figure 3 graphically depicts oscillator settings over time stratified by PARDS severity. It was possible to maintain *F* ≥ 8 Hz and ∆*P*_proximal_ within the target range of 70–90 cmH_2_O, irrespective of age or PARDS severity throughout the study period. After the RM, there was no significant difference in mPaw between the three PARDS severity groups, although children > 60 months required significantly higher mPaw (*p* < 0.001). The mPaw decreased significantly over time (*p* < 0.001). The rate of decrease in mPaw over time was not affected by PARDS severity or age. None of the patients suffered from clinically apparent barotrauma.

### Effect on metrics for oxygenation and ventilation as function of severity

**Fig. 4:**
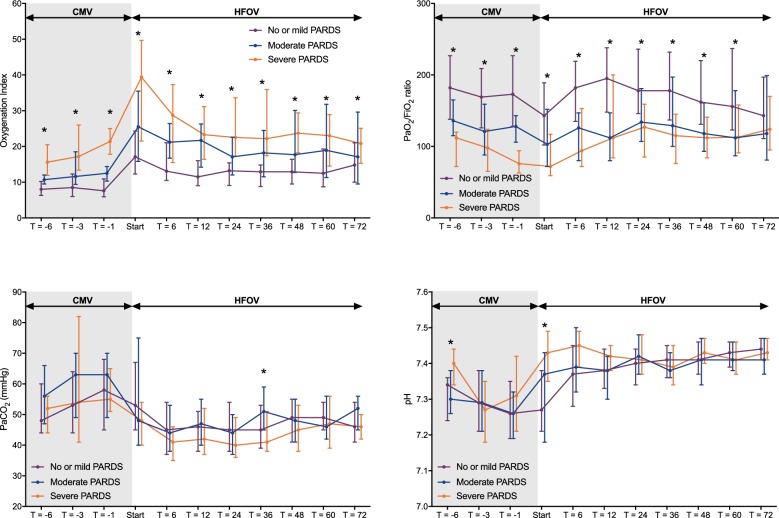
Level and time course of the oxygenation index (OI) (upper left panel), PaO_2_/FiO_2_ ratio (upper right panel), PaCO_2_ (lower right panel) and pH (upper left panel) during the last 6 h of conventional mechanical ventilation (CMV) and the first 72 h of high-frequency oscillatory ventilation (HFOV), stratified by paediatric acute respiratory distress syndrome (PARDS) severity (*N* = 62 children no/mild PARDS, *N* = 29 children moderate PARDS, *N* = 24 children severe PARDS). “Start” is the first measurement immediately after the recruitment manoeuvre. Data are depicted as median (25–75 interquartile range). *p < 0.05

Figure 4 shows the level and time course of oxygenation and ventilation stratified per PARDS severity. The increase in OI immediately after the RM seen in all three groups was caused by an increase in mPaw. The OI decreased in all three groups over time, but this decrease was only significant (*p* < 0.01) in the severe PARDS group. Similarly, the PaO_2_/FiO_2_ ratio improved significantly over time in the severe PARDS group (*p* < 0.01). The PaCO_2_ and pH significantly improved in the first 6 h following the RM (*p* < 0.01), but, thereafter, there was no further change. These changes were similar when stratified by age or PARDS severity, except for the normalization of pH which was more rapid in patients with no/mild PARDS (*p* < 0.01).

### Effect on haemodynamics, fluid management, and organ function as function of age

**Fig. 5:**
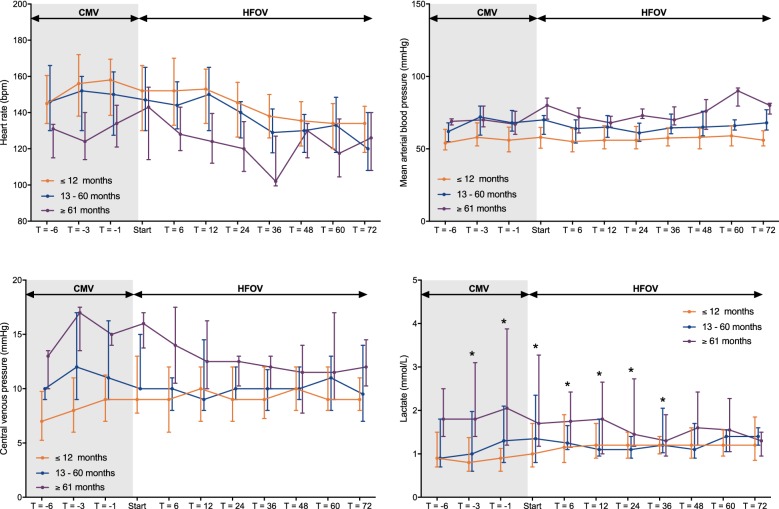
Level and time course of hemodynamic parameters including heart rate (upper left panel), mean arteria blood pressure (upper right panel), central venous pressure (lower left panel) and blood lactate (lower right panel) during the last 6 h of conventional mechanical ventilation and the subsequent first 72 h of high-frequency oscillatory ventilation, stratified by age group (*N* = 83 children ≤ 12 months, *N* = 20 children 13–60 months and *N* = 12 children ≥ 61 months). “Start” is the first measurement immediately after the recruitment manoeuvre. Data are depicted as median (25-75 interquartile range). *p < 0.05

**Fig. 6:**
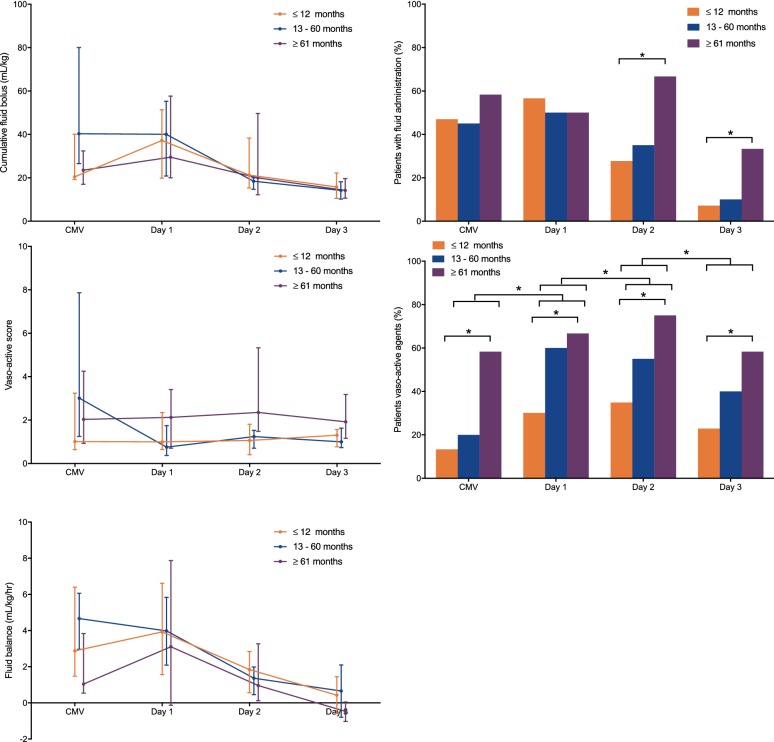
Level and time course of the cumulative amount of fluid boluses in mL/kg (upper left panel), vasoactive score (middle left panel) and the cumulative fluid balance in mL/kg (lower left panel), and the percentage of patients who received fluid boluses per day (upper right panel) and patients on vasoactive support per day (lower right panel), stratified by age group (*N* = 83 children ≤ 12 months, *N* = 20 children 13–60 months and *N* = 12 children ≥ 61 months). Continuous data are depicted as median (25-75 interquartile range) and categorical data as  % of total. CMV conventional mechanical ventilation. *p < 0.05

We did not observe a negative effect over time on HR, mABP, central venous pressure (CVP), or arterial lactate when patients were either stratified by age or PARDS severity (Fig. 5). At the same time, the number of patients who required vasoactive support increased significantly over time (Fig. 6). Yet, the median vasoactive score did not increase significantly over time, suggesting no increase in haemodynamic instability. More children > 60 months were administered a fluid bolus on the second (*p* = 0.027) and third day (*p* = 0.022) of HFOV than younger children, but the cumulative amount of fluid challenges was not significantly different between the age groups (Fig. 6). All of these observations remained the same when stratified for PARDS severity.

The median non-respiratory PELOD-2 score for patients on CMV was 2 (25–75 IQR 0–3). This score remained consistent throughout the first 3 days after transition to HFOV (day 1: median 0 (0–2); day 2: median 2 (0–3); day 3: median 2 (0–3) and was not influenced by of age or PARDS severity.

### Use of sedative-analgesic drugs and NMBA

**Fig. 7:**
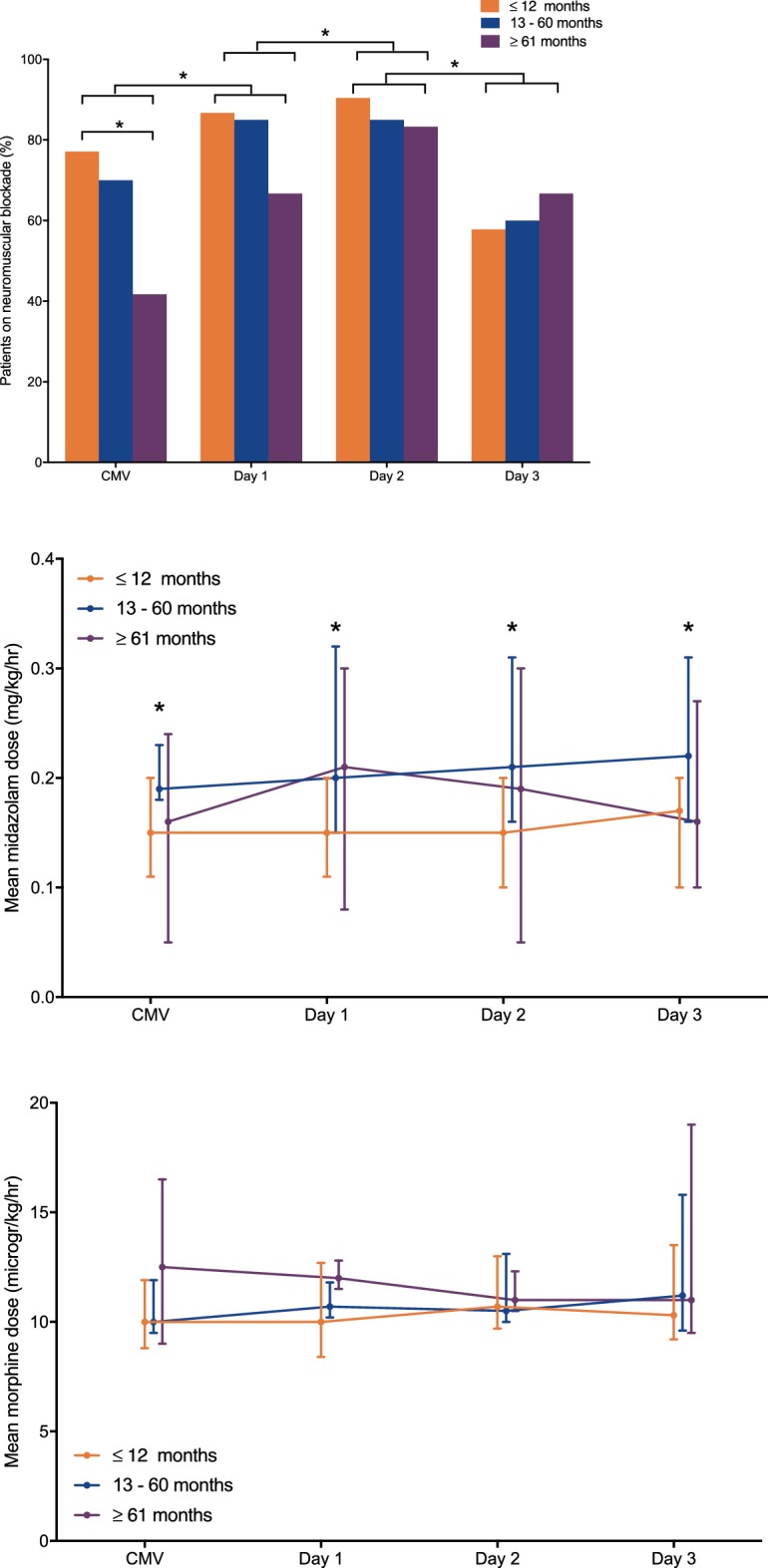
Percentage of patients who were on neuromuscular blocking agents (NMBA) (upper panel), mean midazolam dose (mg/kg/hour) (middle panel) and mean morphine dose (mcg/kg/hour) (lower panel) stratified by age group (*N* = 83 children ≤ 12 months, *N* = 20 children 13–60 months and *N* = 12 children ≥ 61 months). Continuous data are depicted as median (25–75 interquartile range) and categorical data as  % of total. CMV conventional mechanical ventilation. *p < 0.05

We observed a significant increase in NMBA administration in the first 48 h (*p* < 0.01) of HFOV use, after which NMBA use significantly decreased (*p* < 0.01) from day 2 to day 3 (Fig. 7). There was no significant change in the mean midazolam (mg/kg/hr) or morphine (mcg/kg/hr) dosage in any of the age or PARDS severity groups over time (Fig. 6). Children < 12 months required less midazolam than older children when on CMV and during the first 3 days of HFOV (Fig. 7). All of these observations remained the same when stratified for PARDS severity.

## Discussion

To the best of our knowledge, this is the first study reporting that an individualized, physiology-based open-lung HFOV approach characterized by high *F* and high initial ∆*P*_proximal_ in paediatric patients is feasible and does not impair gas exchange or haemodynamics, irrespective of age or PARDS severity.

HFOV is traditionally considered as rescue therapy in case of refractory hypoxemia, although there are some paediatric reports supporting earlier use [6, 20]. Our liberal criteria for transitioning to HFOV may be interpreted as lack of full optimization of CMV. Indeed, we employ HFOV early in the disease trajectory when a patient meets pre-defined criteria rather than using it as a rescue intervention as proposed in international consensus statements in order to prevent ventilator settings from becoming toxic [21, 22]. Inherently, the number of patients managed with HFOV might be higher than in comparable centres including a wider patient spectrum not limited to only patients with severe lung disease. Our liberal use may therefore explain the observed differences in improvement in OI and PaO_2_/FiO_2_ ratio between PARDS severity strata. The greatest improvement in these metrics was observed in patients with severe PARDS, confirming previous observations [20].

Importantly, our study was *not* designed to examine superiority of HFOV over CMV. At the same time, when interpreting our results, it cannot be ruled out that, at least in a proportion of patients in our population, similar effects on oxygenation and ventilation could have been achieved if a (ultra-)lung protective ventilation strategy (i.e., lower Vt in combination with RM) continued, and NMBA administration, prone positioning, and/or higher PEEP were used, potentially in combination. It must be acknowledged that the best strategy to optimize CMV in children with severe PARDS remains uncertain [22]. Recommendations for ventilator management and use of (non-)pulmonary-specific ancillary treatments in PARDS have been made, but these are largely based on expert opinion rather than scientific evidence [21, 23, 24]. Paediatric observational studies have reported use of higher levels of PEEP in PARDS than in our cohort, although it is common among the paediatric critical care community to tolerate higher FiO_2_ [25–29]. To date, there is no specific PEEP strategy shown to be beneficial nor are there RCT data demonstrating that higher PEEP is better than lower PEEP in PARDS, although there are some suggestions that lower PEEP in PARDS may be associated with increased mortality [29]. We also do not know the optimal Vt in (severe) PARDS [30]. To date, prone positioning has not been shown to improve patient outcome in PARDS, although this may also be explained by not restricting its use to severe PARDS [31]. Short-term use of NMBA early in the course of MV has been shown to improve outcome in adults with ARDS, but this remains unclear in paediatrics [32]. Thus, when it comes to paediatric MV, many unknowns remain [33]. Our study underscores the need for systematic testing our HFOV approach on patient outcome. This can only be done in a well-designed RCT.

Although our approach to HFOV may not be considered novel, it is not uniformly used in children [20, 34–37]. It is more custom to set *F* and ∆*P*_proximal_ according to age, mPaw, and observation of chest wiggle [2, 20]. However, such an approach ignores the respiratory system mechanics of the underlying disorder. Furthermore, a RM after switching to HFOV is not routinely performed. We have adopted a physiology-based and individualized approach to HFOV that: a) makes use of a high *F* that allows for sufficient gas exchange and a high initial ∆*P*_proximal_ with a fixed power setting, and b) a staircase incremental–decremental mPaw titration aimed at finding the lowest mPaw on the deflation limb of the P–V loop after switching to HFOV. The rationale behind choosing the highest possible *F* is based on the concept of the corner frequency (*Fc*), which is influenced by the resistance and compliance of the respiratory system [14]. *Fc* identifies the point of the lowest pressure cost of ventilation, i.e. the point that should be the least injurious to the lung. *Fc* is increased when there is reduced Crs, suggesting targeting *F* ≥ 8 Hz [2, 14]. Our data show that we were able to achieve *F* > 8 Hz, irrespective of age or severity of PARDS. We did not find impaired CO_2_ elimination despite these higher *F*. Despite the “high” initial ∆*P*_proximal_, PaCO_2_ and pH levels normalized within 6 hours after transitioning to HFOV, especially in patients with moderate or severe PARDS. Although it cannot be ruled out that normalization of the pH was also partially due to our liberal use of furosemide, our data also suggest that even higher *F* may be used to accept higher PaCO_2_. We arbitrarily choose the initial ∆*P*_proximal_ not to exceed 90 cm H_2_O as higher values may theoretically expose the proximal airways to injurious pressures. Reassuringly, dampening of the pressure swings among the endotracheal tube is much greater when high *F* is used, thereby diminishing the likelihood of proximal airway injury [38].

We always perform a lung optimization manoeuvre after switching to HFOV to optimize end-expiratory lung volume (EELV) as the PaO_2_ is linearly correlated with EELV [39]. Aside from optimizing lung volume to improve oxygenation, pressure oscillations are less dampened in regions with poor compliance, thereby causing larger pressure swings [13]. One group of investigators found that a stepwise mPaw increase produced the greatest increase in lung volume and resolution of atelectasis compared with a 20-s sustained dynamic inflation (either once or repeated 6 times) or a standard approach (i.e. random setting of mPaw) in a neonatal lamb model [40]. We have adopted such a stepwise increase–decrease mPaw titration as a RM strategy. This allows us to individualize the mPaw setting and oscillate on the deflation limb of the P–V loop where there is less continuous recruitment and decruitment of alveoli [41]. Nonetheless, there is a need for comparing various types of RM for determining optimal EELV as the best RM in HFOV still needs to be identified. Furthermore, there appeared to be a slow decrease in mPaw during the first 3 days after start of HFOV. This could mean that we did not wean HFOV aggressively enough, calling for a better protocolization of HFOV weaning instead of a gradual reduction of the mPaw when oxygenation was satisfactorily.

Although the number of patients put on vasoactive drugs increased over time, the vasoactive score (which includes dosages of vasoactive drugs) did not increase. Furthermore, the cumulative amount of fluid boluses remained low in our patient population. This suggests that there was no haemodynamic instability in our study population despite the delivery of a higher mPaw. We did note an increase in NMBA use during the first 48 h of HFOV, especially in children > 60 months, possibly reflecting work of breathing in these patients when breathing spontaneously due to the fixed bias flow of HFOV [42].

A key strength of this study is that we used a protocolized approach to paediatric HFOV in terms of patient selection and management, taking disease trajectory into account. This has not been previously reported. At the same time, there are limitations that should be considered. Although we describe one of the largest cohorts of paediatric HFOV patients, our study represents a single-centre experience with a liberal use of HFOV thereby including a subgroup of patients with less severe disease [2]. Furthermore, practice variability may have influenced our findings, but this is inherent to the study design and can only be overcome by a RCT [43]. Last, from a theoretical perspective, HFOV seems to be a suitable mode for lung-protective ventilation (LPV). The effect of our alternative approach on the development of ventilator-induced lung injury (VILI) cannot be deducted from the present study and warrants further investigations. Our approach is currently being tested in a 2-by-2 factorial randomized controlled trial comparing the effects of ventilation strategy (CMV vs. HFOV) with or without prone positioning on patient outcome (www.prospect-network.org).

## Conclusion

In summary, we report the feasibility in terms of oxygenation, ventilation, and haemodynamics of using an alternative, individualized, physiology-based open-lung approach to HFOV that targets high *F* and high ∆*P*_proximal_. Further research including multisite dissemination and systematic evaluation compared to CMV on both short- and long-term outcomes in paediatric practice is warranted.

## Abbreviations

∆*P*_proximal_: proximal pressure amplitude
AC: assist control
ARDS: acute respiratory distress syndrome
CMV: conventional mechanical ventilation
Crs: compliance of the respiratory system
CVP: central venous pressure
EELV: end-expiratory lung volume
ETT: endotracheal tube
*F*: frequency
*Fc*: corner frequency
HFOV: high-frequency oscillatory ventilation
HR: hear rate
IQR: interquartile range
mABP: mean arterial blood pressure
mPaw: mean airway pressure
NMBA: neuromuscular blocking agent
OI: oxygenation index
P–V loop: pressure volume loop
PARDS: paediatric acute respiratory distress syndrome
PC: pressure control
PEEP: positive end-expiratory pressure
PELOD: paediatric logistic organ dysfunction
PICU: paediatric intensive care unit
PIP: peak inspiratory pressure
PRISM: paediatric risk of mortality
RCT: randomized controlled trial
RM: recruitment manoeuvre
SIMV: synchronized intermittend mandatory ventilation
VIS: vasoactive intrope score
Vte: expiratory tidal volume
